# Traditional Chinese Medicine Body Constitutions and Psychological Determinants of Depression among University Students in Malaysia: A Pilot Study ^†^

**DOI:** 10.3390/ijerph18105366

**Published:** 2021-05-18

**Authors:** Sin Yee Yap, Chai Nien Foo, Yang Mooi Lim, Foong Leng Ng, Sherina Mohd-Sidik, Pek Yee Tang, Jagjit Kaur Najar Singh, Kai-Shuen Pheh

**Affiliations:** 1 Centre for Cancer Research, Faculty of Medicine and Health Sciences, Universiti Tunku Abdul Rahman, PT21144, Jalan Sungai Long, Bandar Sungai Long, Kajang 43000, Selangor, Malaysia; sinyeeyap24@gmail.com (S.Y.Y.); ymlim@utar.edu.my (Y.M.L.); ngfl@utar.edu.my (F.L.N.); jagjitk@utar.edu.my (J.K.N.S.); 2 Department of Population Medicine, Faculty of Medicine and Health Sciences, Universiti Tunku Abdul Rahman, PT21144, Jalan Sungai Long, Bandar Sungai Long, Kajang 43000, Selangor, Malaysia; 3 Department of Pre-Clinical Science, Faculty of Medicine and Health Sciences, Universiti Tunku Abdul Rahman, Lot PT21144, Jalan Sungai Long, Bandar Sungai Long, Kajang 43000, Selangor, Malaysia; 4 Department of Traditional Chinese Medicine, Faculty of Medicine and Health Sciences, Universiti Tunku Abdul Rahman, PT21144, Jalan Sungai Long, Bandar Sungai Long, Kajang 43000, Selangor, Malaysia; 5 Department of Psychiatry, Faculty of Medicine and Health Sciences, Universiti Putra Malaysia, UPM Serdang, Seri Kembangan 43400, Selangor, Malaysia; sherina@upm.edu.my; 6 Department of Mechatronics and Biomedical Engineering, Lee Kong Chian Faculty of Engineering and Science, Universiti Tunku Abdul Rahman, Lot PT21144, Jalan Sungai Long, Bandar Sungai Long, Kajang 43000, Selangor, Malaysia; tangpy@utar.edu.my; 7 Department of Nursing, Faculty of Medicine and Health Sciences, Universiti Tunku Abdul Rahman, PT21144, Jalan Sungai Long, Bandar Sungai Long, Kajang 43000, Selangor, Malaysia; 8 Department of Psychology and Counselling, Faculty of Arts and Social Science, Universiti Tunku Abdul Rahman, Jalan Universiti, Bandar Barat, Kampar 31900, Perak, Malaysia; phehks@utar.edu.my

**Keywords:** depression, traditional Chinese medicine, body constitution, psychological factors

## Abstract

Depression is commonly observed in university students, who are a high risk group for developing psychiatric disorders during adulthood. This study aimed to determine the prevalence of depression and its traditional Chinese medicine body constitutions and psychological determinants among university students in Malaysia. A cross-sectional pilot study was conducted between 9 and 28 September 2020 among 80 university students in Malaysia. Participants completed online survey questionnaires, including the validated Patient Health Questionnaire (PHQ-9), Constitution in Chinese Medicine Questionnaire (CMCQ), Dysfunctional Attitude Scale (DAS), Depression Anxiety Stress Scale (DASS-21) stress subscale, Perceived Stress Scale (PSS-10), and Rosenberg Self-Esteem Scale (RSES), which assess depression, body constitution, dysfunctional attitude, stress, perceived stress, and self-esteem. Multiple linear regression analyses were performed to determine the associated risk factors for depression. The overall prevalence of depression among university students was 33.8%. The multiple regression analysis showed a significant relationship between depression and qi-stagnation constitution (B = 0.089, *p* = 0.011), balanced constitution (B = −0.077, *p* = 0.049), and self-esteem (B = −0.325, *p* = 0.001). Our findings suggest that some traditional Chinese medicine body constitutions and self-esteem are significant risk factors affecting depression among university students. Identifying risk factors of depression is vital to aid in the early detection of depression among university students.

## 1. Introduction

Depression is a major contributor to the overall global burden of disease [1,2]. To date, depression is estimated to affect 264 million people of all ages worldwide [3]. Depression often develops at a young age; such a disorder can affect people’s functioning and is often recurring [1]. Depressive symptoms include persistent sadness, loss of interest, decreased concentration or energy, disturbed appetite or sleep, and strong feelings of worthlessness or guilt [4]. These symptoms are recurring and can interfere with a person’s ability to take care of their everyday responsibilities [1]. When left untreated, depression may cause severe health conditions. For instance, people who are depressed are more vulnerable to cardiovascular disease [5,6]. At its worst, depression can increase a person’s risk of suicide [1].

University students are a high at-risk group for developing depression and suicidal ideation [7]. The prevalence of depression among university students in Malaysia is relatively high and alarming, which has been found to be between 14.0% to 30.7% in past studies [8,9,10,11,12,13]. Studies conducted among universities in urban areas such as Klang Valley (between 27.5% to 29.3%) [8,9] and Melacca (30.7%) [12] had higher prevalence rates of depression compared with studies conducted in a rural area like Sarawak (14.0%) [13]. This is consistent with past studies, which showed that the risk for depression is generally higher in cities than in rural areas [14,15]. Various factors, including higher stress exposure [16], increased pollution [14], reduced time spent with family [17], lack of physical activity [17], higher violence, and crime rates [15], may contribute to the development of depression among university students in cities. For example, students who live in the city may have less access to nature, reduced social support, higher noise levels, and air pollution. As these factors erode, these students may become more vulnerable to depression.

Body constitution is the foundation of traditional Chinese medicine (TCM). It focusses on what makes an individual vulnerable to specific illness [18]. Each individual has a unique body constitution [19,20]. Our body constitution can be changed over time and by acquired factors such as diet, lifestyles, and environment [21]. There are nine types of TCM body constitutions. First is the balanced constitution (BC), which is a neutral constitution, along with the following eight types of unbalanced constitutions: qi-stagnation (QSC), blood-stasis (BSC), qi-deficiency (QDC), yang-deficiency (YADC), yin-deficiency (YIDC), phlegm-dampness (PDC), damp-heat (DHC), and inherited-special (ISC) [20]. The classification and determination of each TCM body constitution is based on an individual’s physical features, psychological characteristics, physiological characteristics, susceptibility to certain illnesses, and adaptability to the environment [20,22].

People with BC generally have a strong physique, and are emotionally and mentally stable as they have harmony of yin–yang, qi, and blood in their body [20]. They are adaptable to environmental changes and do not suffer from illness easily [22]. People with the qi dynamic disturbed in their body are categorized as QSC; they are mostly thin and tend to be suspicious [20]. They also respond to stressful situations poorly [23]. Next, people with BSC are often forgetful and impatient [20]. They tend to feel uncomfortable in cold environments and are vulnerable to bleeding and body pain caused by obstructed blood flow within the body [20]. Generally, people with QDC are introvert and timid in personality; they catch colds or flu easily and are sensitive to environmental changes [20]. They often require a longer time for recovery because of their weak immune systems [24]. People with YADC are mostly quiet, sleepy, and introverted [20,24]. These individuals often have cold hands and feet and they are sensitive to low temperatures [24]. Conversely, people with YIDC usually are thin, outgoing, and impatient [20]. These individuals usually have warm palms and soles, a preference for cold drinks, and feel uncomfortable in hot and dry environments [24]. People with PDC are often overweight, and have mild temperaments and patience [22,24]. They feel uncomfortable in humid and rainy environments [22]. People with DHC have an average or thin physique [20] and are irritable and sensitive to humid and hot environments [20]. Lastly, people with ISC usually have inborn weakness [20]. These people are susceptible to skin eruptions, drug allergies, asthma, seasonal changes, and other environmental allergens [24]. A larger proportion of unbalanced body constitution types have been found in patients who suffered from depression than those with a balanced body constitution [23,25,26,27,28,29].

Psychological factors associated with depression include dysfunctional attitude, stress, perceived stress, and self-esteem. It has been well documented that dysfunctional attitudes are related to mood disorders [30,31,32,33,34,35]. Dysfunctional attitudes are beliefs and attitudes that induce negative thoughts about oneself, others, and the future [36]. The findings obtained in studies have shown that depressed groups in adolescents and youth are closely related to the selective processing of negative information [37]; negative attributional style [33,38]; overgeneralizing; selective abstraction; and negative evaluation of the self, world, and future [34,35,39,40].

Stress is our body’s response to pressures from a situation or life event [41,42]. During university, students are exposed to various stressful experiences, ranging from academic [43,44], personal [45,46], to social stress [46,47]. These ongoing stresses can negatively affect students’ learning capacity, academic performance, education, employment attainment, sleep quality and quantity, physical health, mental health, and substance use outcomes [48]. Previous studies among undergraduate students have shown that psychological stress is significantly associated with depression [49,50]. Psychological stress can induce a series of physiological responses in the nervous, endocrine, and immune systems, which can be harmful under certain conditions [51,52,53].

On the other hand, some findings have shown that depression might be correlated with perceived stress [33,54,55,56]. Unlike stress, perceived stress is more about a person’s feelings about lack of control and unpredictability rather than the actual stressors [57]. Pozzebon et al. [54] demonstrated that the severity of depression is affected by the level of perceived stress. According to previous studies [16,58], students may find it hard to manage the increasing workload or fear of failing exams during university. As these negative thoughts accumulate, their perceived stress level may increase.

Additionally, the development of depression has been associated with the low level of self-esteem [35,59,60]. Self-esteem is a person’s overall sense of his or her value or worth [61]. It can be considered as a measure of how much an individual “values, appreciates, or likes him or herself” [62]. Research has demonstrated that people with a lower level of self-esteem are more prone to high risk behaviours or inappropriate coping styles, increasing the risk of depression [63].

By identifying the risk factors related to depression, it is possible to prevent depression; we can make changes to our diets, learn to cope with stress, and maintain a healthier lifestyle. Therefore, this pilot study aimed to examine the weighted prevalence of depression and to provide insight into the correlations of depression among university students in Malaysia. It is hypothesized that certain TCM body constitutions and the effects of psychological factors, such as dysfunctional attitude, stress, perceived stress, and self-esteem, may contribute to the development of depression.

## 2. Materials and Methods

A cross-sectional pilot study online survey was conducted between 9 and 28 September 2020. The data were collected via Google Forms using snowball sampling. University students aged between 18 and 30, of Malaysian nationality, studying in Malaysia, and who agreed to participate were recruited. The sample size of this pilot study was calculated based on the one in ten rule, where one predictive variable is applied for every ten events [64,65,66]. Ten samples were recruited for each variable: (1) depression, (2) TCM body constitution, (3) dysfunctional attitude, (4) stress, (5) perceived stress, and (6) self-esteem, with the dropout rate of 25.0% [67]. The calculation formula was as follows: (10 × number of variables) ÷ (1–0.25). Thus, the final estimated sample size for this pilot study was 80. This study was approved by the Universiti Tunku Abdul Rahman Ethics Committee for research involving human subjects, prior to the commencement of the study (U/SERC/50/2020). Written consent forms were obtained from all of the subjects. The data collected were kept private and anonymous.

The survey questionnaires used in this study included the pre-tested and validated Patient Health Questionnaire (PHQ-9) [68], Constitution in Chinese Medicine Questionnaire (CMCQ) [69], Dysfunctional Attitude Scale (DAS) [70], Depression Anxiety Stress Scale (DASS-21) Stress Subscale [71], Perceived Stress Scale (PSS-10) [57], and Rosenberg Self-Esteem Scale (RSES) [72] to assess depression, body constitution, dysfunctional attitude, stress, perceived stress, and self-esteem. Depression was measured using PHQ-9, because it has equivalent validity to the Diagnostic and Statistical Manual of Mental Disorders, fifth Edition (DSM-5), and showed a high sensitivity of 88% and high specificity of 85% [73]. The socio-demographic characteristics and health status of the participants were recorded.

Statistical Package for Social Sciences version 25 (SPSS V.25.0) (IBM, Armonk, NY, USA) was used to analyze the data. Chi-square was performed to investigate the associations of TCM body constitution, dysfunctional attitude, stress, perceived stress, and self-esteem with depression. Fisher’s exact test was referred to when the expected frequency for a particular category was more than 20%. The relationships between risk factors and depression were then analyzed using multiple linear regressions. All of the variables with a *p* value less than 0.05 were entered into the multiple linear regression model. The enter method was used. All of the tests were two-tailed, and the significance level (α) was set at a *p* value of <0.05.

## 3. Results

### 3.1. Socio-Demographic Characteristics and Health Status

Table 1 shows the socio-demographic characteristics and health status of the subjects. Twenty-four (30.0%) of the subjects were males, and fifty-six (70.0%) were females. The majority of subjects were Chinese (87.5%) and were in the age group of 21 and 23 years old (81.3%). A total of sixty-eight out of eighty subjects were currently pursuing undergraduate degrees, while five and seven subjects were pursuing foundation and postgraduate degrees, respectively. 13.8% of the subjects had a history of smoking, while 3.8% reported being current smokers. For alcohol consumption, 62.5% reported drinking once monthly or less, followed by 21.3% who never drink, 13.8% who drink two to four times a month, 1.3% who drink two to three times a week, and 1.3% who drink four or more times in a week. Most subjects did not have a history of health problems (70.0%) or mental health problems (87.5%). Ten (12.5%) subjects reported having a family history of depression.

### 3.2. Depression, Traditional Chinese Medicine Body Constitutions, Dysfunctional Attitude, Stress, Perceived Stress, and Self-Esteem

The overall mean score of PHQ-9 was 8.0 (SD = 5.4). According to the PHQ-9 cut off points ≥10, about 33.8% (*n* = 27) of subjects were shown to have depression (Table 2), with a greater prevalence among females (26.3%) than males (7.5%). Fourteen (17.5%) of the subjects had a balanced constitution, while sixty-six (82.5%) showed unbalanced or mixed constitutions. Among the unbalanced body constitution types, blood-stasis (17.5%) recorded the highest, followed by qi-deficiency (15.0%), inherited-special (11.3%), qi-stagnation (10.0%), yin-deficiency (8.8%), mixed constitutions (7.5%), yang-deficiency (6.3%), damp-heat (3.8%), and phlegm-dampness (2.5%). About 17.5% of the subjects were shown to have dysfunctional attitudes. A high stress level was reported among 23.8% (*n* = 19) of the subjects. The majority of subjects (87.5%) reported experiencing perceived stress. For self-esteem, 50.0% of the subjects reported having a low level of self-esteem.

### 3.3. Association Analysis between Variables

A chi-square test of independence showed that there were significant associations between depression and TCM body constitutions (*p* = 0.03), dysfunctional attitude (*p* < 0.001), stress (*p* < 0.001), perceived stress (*p* = 0.01), and self-esteem (*p* < 0.001; Table 3).

### 3.4. Multiple Linear Regression Analysis

A multiple linear regression analysis was performed to examine the relationships between depression and various potential predictors. The multiple regression model with all six predictors produced R^2^ = 0.740, F (6, 73) = 34.71, *p* < 0.001. Details of the analysis results are shown in Table 4. Qi-stagnation constitution (B = 0.089, *p* = 0.011) was positively and significantly associated with depression, indicating that the subjects with qi-stagnation constitution tend to have a higher risk of depression. Balanced constitution (B = −0.077, *p* = 0.049) and self-esteem (B = −0.325, *p* = 0.001) were negatively associated with depression, indicating that subjects with balanced constitution or high self-esteem have a lower risk of depression.

## 4. Discussion

### 4.1. Depression, Traditional Chinese Medicine Body Constitutions, Dysfunctional Attitude, Stress, Perceived Stress, and Self-Esteem Levels among University Students

The prevalence rate of depression among university students was 33.8%; among them, females have been shown to have a high risk of depression. The prevalence rate of depression in this study was higher than in previous similar research conducted among university students in public and private universities in the Klang Valley [8,9,10,11], which ranged between 21.0% and 30.0%. Given the current COVID-19 pandemic outbreak in Malaysia, the increase in depression might due to changes in daily lifestyles [74].

The higher proportion of females could be due to the unequal numbers of male and female subjects in the study, and this reflects the gender proportion in the current higher education sector where females made up 60.0% and 51.0% of the total enrolment in public and private higher education institutions, respectively [75]. However, many studies have also demonstrated that depression is more common in females than males [76,77,78]. Given that females experience the peak onset of depressive episodes during their reproductive years [79,80], the hormonal difference may have a role in increasing the risk of depression among females. Furthermore, females are generally socialized to be more sensitive to the opinions of others [81,82], and they tend to mull their problems over in their minds [77,83]. This ruminative coping style may explain why they are more vulnerable to depression. The depression gender gap is not due to biology alone. Life stressors and culture can contribute to the occurrence of depression too. Females usually have more social roles than males, making them suffer from excessive workloads [84]. Moreover, sociocultural forces may be among the contributing factors to the gender disparity of depressive disorders. In particular, Malaysians suffer from the worst gender inequity among Southeast Asian countries, with a World Economic Forum’s Global Gender Gap Index of 0.68 [85]. Several recent studies have documented the disastrous consequences of gender inequality on women’s mental health [86,87,88], as observed in our study. Prompt actions are needed in order to achieve gender justice in Malaysia, so as to address the burden of depression and other public health problems. 

The findings of the present study showed that 82.5% of the subjects had unbalanced constitutions. Blood-stasis constitution was the most frequent unbalanced body constitution type among university students in the present study, followed by qi-deficiency. According to TCM, people with BSC usually have an obstructed, or even stagnant, blood circulation, while people with QDC often have insufficient qi and weak immunity [19]. An empirical study conducted in 2019 revealed that the prevalence of overweight and obesity among Malaysian university students was 21.2% and 16.3%, respectively [89]. One of these factors could be the reason BSC was frequently detected in the present study. Overweight and obese students are at risk of many severe health conditions, including hypertension [90,91] and high cholesterol [92], which can affect blood circulation and result in blood stagnation. Furthermore, previous studies have shown that BSC is closely related to primary dysmenorrhea among young females [93,94]. As most of the present study subjects were women, this could explain why BSC was more commonly detected.

University students usually have inadequate sleep beause of their addiction to smartphones [95]. A study on Korean university students showed that smartphone addiction is significantly correlated to a lack of sleep [96]. According to TCM, our qi draws inward to restore our body during sleep [19]. Poor sleep quality may affect our organs’ maintenance schedule, leading to an insufficient supply of qi and blood, inducing QDC [19]. Moreover, physical inactivity could be one of the risk factors for BSC and QDC, as a lack of movement may affect our blood circulation. A recent systematic review found a significant reduction in physical activity levels among university students because of the COVID-19 pandemic [97]. The pandemic outbreak and the resulting lockdowns may have impacted university students’ ability to be physically active. The opportunities for them to participate in sports and exercises might have been restricted or temporarily eliminated. In TCM, the impedance of blood flow may affect the qi-blood circulation in our body, leading to the formation of unbalanced constitutions like BSC and QDC [19].

Dysfunctional attitudes were found among 17.5% of the subjects, with a higher proportion among females. The presence of dysfunctional attitudes in the current study could be because these students have high expectations towards their academic and social performance, and can become overly critical with themselves when they fail to fulfill such expectations [98]. The academic challenges, concerns for the future, and financial burdens may also contribute to the development of dysfunctional attitudes among subjects [99]. Among the factors measured by DAS, perfectionism [100] was the most detected among the current finding subjects, which is consistent with previous studies conducted among Malaysian tertiary students [98,101]. The higher level of dysfunctional attitudes detected in females could be because females subjects are more sensitive and have greater perfectionist attitudes than males [99,102].

Stress was detected in 23.8% of the subjects, consistent with previous similar studies conducted among university students [8,9,103]. The elevated stress level could be due to the impact of the transitional and adaption periods to the new online learning mode implemented during the pandemic [104,105]. University students experiencing increased stress during the pandemic might have been due to increased concerns over grades and delayed graduation [104]. Some students may have also experienced higher stress levels because of financial difficulties and uncertainty regarding future employment, as the pandemic significantly reduced global economic production and increased unemployment [104,106]. Moreover, pandemic-related uncertainty and health concerns may have contributed to increased stress [104,107].

Perceived stress was shown to be prevalent (87.5%) among university students in the present investigation. This is higher than in a previous study conducted among adults in Selangor, Malaysia (54.3%) [16]. Stressors among university students are often related to exams, academics, relationship problems, and financial problems [45,47]. Isolation experienced during the lockdowns could have affected these students’ opportunities to seek support [108]. Consequently, these ongoing and unresolved negative thoughts may have contributed to the increased perceived stress level.

About half of the subjects were reported to have low self-esteem in the present findings. This could be due to the higher proportion of Chinese subjects in the present study. Asian students are more commonly reported with lower self-esteem compared to other ethnicities such as Western students [109]. There are a few factors that could contribute to the self-esteem gap between Asians and other ethnicities. Asians, especially Chinese, parents tend to employ strict and harsh parenting styles focused on achievement, while Western parenting is more permissive [110]. This tiger parenting could contribute to low self-esteem and other mental health outcomes among Chinese youths [111]. Chinese parents often expect their children to have an outstanding academic performance. When these students fail to achieve parents’ ideal standards, they may feel anxious and lack confidence [112].

### 4.2. Relationship between Traditional Chinese Medicine Body Constitutions and Depression

The present study found that qi-stagnation was positively and significantly associated with depression among university students, which is in accordance with similar studies focused on university students [28,29]. Qi-stagnation symptoms included emotionally unstable, easily worried, frequently sighing, low spirit, and low mood [19]. Qi can become stagnated as a result of excessive thought, anxiety, and stress [19]. In TCM, the liver is in charge of dispersion and dredging in order to regulate qi’s movement throughout the body and emotions [19]. Students may experience excessive stress and anxiety during university as they struggle with their studies, experience financial pressure, and are concerned for their future. These factors could affect the function of the liver, resulting in interruptions of qi and blood, which can lead to a variety of problems, including mood swings and depression [19]. Additionally, poor sleep quality and insomnia in university students [113] could influence the liver’s blood storing ability, thus affecting its dispersion and dredging function, which will then cause qi-stagnation [19]. Dysregulation of the liver qi is the main cause of depression, and is associated with the neuroendocrine system [114]. Preliminary studies in Western medicine support that dysregulation of the norepinephrine system located in the locus coeruleus (LC/NE) and the hypothalamic–pituitary–adrenal axis (HPA) might be a root cause for depression [114,115].

Balanced constitution is negatively associated with depression, which means university students with balanced constitution are less likely to develop depression. Students with balanced constitution have balanced yin–yang, qi, and blood in their bodies [19]. They have stable emotional or mental states and are adaptable to environmental changes [19]. In TCM, individuals’ body constitutions reflect the power of their bodies’ disease resistance—physically and mentally [18]. Students with balanced constitution are often able to withstand pathogen invasion. Even if these students are infected with disease, their conditions are usually less severe and can be easily cured in a short time [18]. Additionally, these students have relatively healthier lifestyles compared with others with unbalanced constitutions [19]. Previous research has demonstrated that lifestyle changes can enhance mood and mental health [74]. Therefore, understanding the concept of body constitution can help prevent and treat depression.

### 4.3. Relationship between Self-Esteem and Depression

People with low self-esteem are more likely to feel sad and lonely. Furthermore, they are more sensitive to rejection. Generally, self-esteem is malleable during adolescence and becomes relatively stable across one’s life [116], indicating that self-esteem in youth may have a critical role in determining the risk of depression during later life stages. Students with low self-esteem may develop high-risk behaviors such as alcohol consumption and marijuana use as a result of peer pressure [63]. Engaging in these health compromising behaviors could contribute to the development of mental illnesses, including depression.

### 4.4. Limitations of the Study

The limitation of the representativeness of the study sample is because of the sampling method used. The findings cannot be generalized to the whole Malaysian university population because of the small sample size. A more detailed study will be conducted using larger sample size and probability sampling method in order to confirm the current findings.

## 5. Conclusions

The prevalence of depression found in the present study is relatively high. As for predictors of depression, qi-stagnation constitution is a motivator for the occurrence of depression. In contrast, balanced constitution and high self-esteem are inhibitors of the development of depression. Addressing mental health during and after this global health crisis should be done by increasing public health concerns in university students.

## Figures and Tables

**Table 1:** Demographic and health status of subjects.

Characteristics	*n*	Percentage (%)
Gender		
Male	24	30.0
Female	56	70.0
Age group		
≤20	8	10.0
21–23	65	81.3
≥23	7	8.8
Ethnicity		
Chinese	70	87.5
Malay	5	6.3
Indian	5	6.3
Education level (current)		
Foundation	5	6.3
Undergraduate	68	85.0
Postgraduate	7	8.8
History of smoking		
Yes	11	13.8
No	69	86.3
Smoker (current)		
Yes	3	3.8
No	77	96.3
Alcohol consumption		
Never	17	21.3
Once monthly or less	50	62.5
2–4 times a month	11	13.8
2–3 times a week	1	1.3
4 or more times a week	1	1.3
History of health problem		
Yes	24	30.0
No	56	70.0
History of mental health problem		
Yes	10	12.5
No	70	87.5
Family history of depression		
Yes	10	12.5
No	70	87.5

**Table 2:** Prevalence of depression, traditional Chinese medicine body constitutions, dysfunctional attitude, stress, perceived stress, and self-esteem among subjects.

Variables	Mean	Standard Deviation	*n*	Percentage (%)
Presence of depression (PHQ-9) (0–27)	8.0	5.4		
Yes (≥10)			27	33.8
No (1–9)			53	66.3
TCM body constitution (CMCQ) (0–100)				
Balanced (0–100)	56.4	13.2	14	17.5
Qi-stagnation (0–100)	37.4	13.3	8	10.0
Blood-stasis (0–100)	38.9	16.3	14	17.5
Qi-deficiency (0–100)	40.9	13.6	12	15.0
Yang-deficiency (0–100)	30.1	16.0	5	6.3
Yin-deficiency (0–100)	36.8	14.2	7	8.8
Phlegm-dampness (0–100)	28.7	15.3	2	2.5
Damp-heat (0–100)	26.4	13.6	3	3.8
Inherited-special (0–100)	31.4	16.8	9	11.3
Dysfunctional attitude (DAS) (19–133)	55.3	17.2		
Yes (>72.54)			14	17.5
No (<72.54)			66	82.5
Stress (DASS-21) (0–42)	10.6	8.4		
High (≥15)			19	23.8
Low (0–14)			61	76.3
Perceived stress (PSS-10) (0–40)	19.6	5.9		
High (14–40)			70	87.5
Low (0–13)			10	12.5
Self-esteem (RSES) (0–40)	26.6	5.1		
High (>26.58)			40	50.0
Low (<26.58)			40	50.0

Abbreviations: PHQ-9, Patient Health Questionnaire; CMCQ, Constitution in Chinese Medicine Questionnaire; DAS, Dysfunctional Attitude Scale; DASS-21, Depression Anxiety Stress Scale stress subscale; PSS-10, Perceived Stress Scale; RSES, Rosenberg Self-Esteem Scale. A higher score indicates a higher level of depression, traditional Chinese medicine (TCM) body constitutions, dysfunctional attitude, stress, perceived stress, and self-esteem. TCM body constitutions are categorized as follows: a score ≥ 60 in Balanced constitution section and scores < 30 in other sections is considered as balanced constitution; while for unbalanced body constitutions (qi-stagnation, blood-stasis, qi-deficiency, yang-deficiency, yin-deficiency, phlegm-dampness, damp-heat, inherited-special), a higher score in a specific body constitution section indicates a higher likelihood of that specific constitution type, a score of 30 is set as the threshold for case definition.

**Table 3:** Associations of traditional Chinese medicine body constitutions, dysfunctional attitude, stress, perceived stress, and self-esteem with depression.

Variables	Presence of Depression		Absence of Depression		*p* Value
	*n*	Percentage (%)	*n*	Percentage (%)	
TCM body constitution (0–100)					0.027 ^a,^*
Balanced	1	1.3	13	16.3	
Unbalanced	26	32.5	40	50.0	
Dysfunctional attitude (19–133)					<0.001 ^a,^*
Yes (>72.54)	11	13.8	3	3.8	
No (<72.54)	16	20.0	50	62.5	
Stress (0–42)					<0.001 *
High (≥15)	16	20.0	3	3.8	
Low (0–14)	11	13.8	50	62.5	
Perceived stress (0–40)					0.014 ^a,^*
High (14–40)	27	33.8	43	53.8	
Low (0–13)	0	0	10	12.5	
Self-esteem (0–40)					<0.001 *
High (>26.58)	5	6.3	35	43.8	
Low (<26.58)	22	27.5	18	22.5	

* significant at *p* < 0.05. ^a^ = Fisher’s exact test. A higher score indicates a higher level of traditional Chinese medicine (TCM) body constitutions, dysfunctional attitude, stress, perceived stress, and self-esteem.

**Table 4:** Summary statistics and results from the regression analysis.

Variables	Simple Linear Regression			Multiple Linear Regression		
	B	95% CI	*p* Value	B	95% CI	*p* Value
TCM body constitution (CMCQ)						
Qi-stagnation (0–100)	0.177	(0.096, 0.259) *	<0.001 *	0.089	(0.021, 0.156) *	0.011 *
Blood-stasis (0–100)	−0.035	(−0.107, 0.038)	0.343			
Qi-deficiency (0–100)	0.011	(−0.099, 0.121)	0.845			
Yang-deficiency (0–100)	−0.051	(−0.120, 0.017)	0.140			
Yin-deficiency (0–100)	−0.027	(−0.112, 0.057)	0.524			
Phlegm-dampness (0–100)	0.023	(−0.056, 0.103)	0.562			
Damp-heat (0–100)	0.003	(−0.076, 0.082)	0.943			
Inherited-special (0–100)	0.018	(−0.042, 0.078)	0.545			
Balanced (0–100)	−0.230	(−0.338, −0.121) *	<0.001 *	−0.077	(−0.155, 0.000) *	0.049 *
Dysfunctional attitude (DAS) (19–133)	0.188	(0.130, 0.247) *	<0.001 *	0.036	(−0.016, 0.087)	0.174
Stress (DASS−21) (0–42)	0.459	(0.353, 0.566) *	<0.001 *	0.117	(−0.002, 0.236)	0.053
Perceived stress (PSS-10) (0–40)	0.656	(0.504, 0.809) *	<0.001 *	0.091	(−0.096, 0.278)	0.334
Self-esteem (RSES) (0–40)	−0.776	(−0.947, −0.604) *	<0.001 *	−0.325	(−0.518, −0.131) *	0.001 *

* significant at *p* < 0.05; higher score indicates higher level of traditional Chinese medicine (TCM) body constitutions (qi-stagnation, blood-stasis, qi-deficiency, yang-deficiency, yin-deficiency, phlegm-dampness, damp-heat, inherited-special, and balanced), dysfunctional attitude, stress, perceived stress, and self-esteem.

## Data Availability

Not applicable.
